# Transformer enhanced based YOLOv8 integration: a hybrid deep learning framework for intelligent insulator defect detection in high-voltage transmission systems

**DOI:** 10.3389/frai.2025.1732616

**Published:** 2026-03-02

**Authors:** Umer Farooq, Fan Yang, Jamshed Ali Shaikh

**Affiliations:** 1 State Key Laboratory of Power Transmission Equipment, Systems Security and New Technology, School of Electrical Engineering, Chongqing University, Chongqing, China; 2 School of Computer Science and Engineering, South China University of Technology, Guangzhou, Guangdong, China

**Keywords:** deep learning, insulator defect detection, power transmission, transformer, YOLOv8

## Abstract

Insulators are vital components of high-voltage power transmission systems, where undetected defects can lead to catastrophic failures and significant economic losses. Accurate and timely detection of insulator defects (IDs) under diverse environmental conditions is critical for ensuring system reliability. This study presents Transformer-Enhanced YOLOv8 (TE-YOLOv8), a novel hybrid deep learning framework designed to address the challenges of detecting small, complex defects in transmission line inspections. TE-YOLOv8 integrates transformer-based attention mechanisms with the advanced YOLOv8 architecture, introducing several key innovations that enhance its performance. Specifically, it incorporates Global Convolution (GConv) modules to capture extended spatial context for improved feature extraction, C3f-Global Pooling Fusion (C3f-GPF) modules to amplify discriminative features, and Multiscale Information Fusion (MSIF) modules with learnable weights for adaptive multi-scale detection. Additionally, it utilizes Weighted Feature Information Fusion (WFIF) modules for channel-wise attention to refine feature representation, and a Transformer-enhanced neck architecture to model global dependencies and provide enhanced contextual understanding. To improve localization precision and accelerate convergence, the framework adopts the SCYLLA-IoU (SIoU) loss function. Extensive experimental validation on the IDID and CPLID datasets demonstrates that TE-YOLOv8 achieves mean average precision (mAP) scores of 94.2% and 93.8%, respectively, representing improvements of 4.9% and 5.1% over the baseline YOLOv8, and 1.9% and 2.0% over TE-YOLOV8, while maintaining real-time inference at 82 frames per second. Ablation studies, precision-recall curves, and visualization analyses further confirm the effectiveness of TE-YOLOv8 in detecting insulator defects under challenging operational conditions.

## 1 Introduction

Electrical power transmission systems constitute the backbone of modern infrastructure, where high-voltage transmission lines spanning vast geographical regions deliver electricity from generation facilities to consumption centers (Yuan et al., 2021). Within this critical infrastructure, insulators play an indispensable role in maintaining electrical isolation between energized conductors and supporting structures, thereby ensuring system safety and operational continuity (Zhou et al., 2022; Liu et al., 2021; Huang et al., 2023). The deterioration or failure of these components can precipitate cascading failures with devastating consequences, including widespread power outages affecting millions of consumers, substantial economic losses exceeding billions of dollars, and severe threats to public safety through electrocution hazards and fire incidents (Yuan et al., 2021; Girshick et al., 2014). Statistical analyses from power utilities worldwide indicate that insulator-related failures account for approximately 35% of all transmission line faults, underscoring the paramount importance of proactive defect detection and preventive maintenance strategies (Girshick, 2015; Ren et al., 2015).

**Figure 1:**
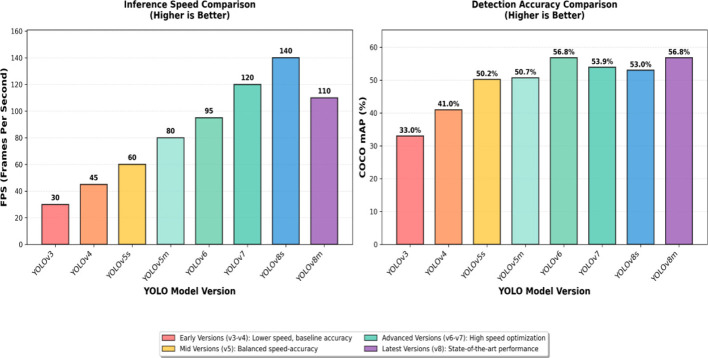
Performance comparison of YOLO series algorithms (YOLOv5 through YOLOv8) on the COCO dataset, demonstrating progressive improvements in detection accuracy, with YOLOv8 achieving superior performance while maintaining real-time inference capabilities.

Traditional manual inspection methodologies suffer from multiple critical limitations, including labor intensity, safety risks, subjective assessment variability, and intermittent scheduling that creates temporal gaps during which defects may progress undetected (Shuang et al., 2023; Ou et al., 2023; Wang et al., 2021). The advent of unmanned aerial vehicle technology has revolutionized transmission line inspection paradigms, enabling systematic aerial surveillance with high-resolution optical sensors (Yang and Wang, 2023; Zhang et al., 2023; Yu et al., 2023). However, the manual processing of extensive image datasets represents a critical bottleneck, motivating the development of automated computer vision systems (Hao et al., 2022; Wang et al., 2020). Recent advances in deep learning, particularly the YOLO family of object detection algorithms, have demonstrated remarkable capabilities in real-time detection applications (Redmon et al., 2016; Redmon and Farhadi, 2018; Bochkovskiy et al., 2020; Li et al., 2022; Wang et al., 2023). Insulator defect detection presents unique challenges, including extreme scale variation, complex background clutter, diverse defect morphologies, and adverse imaging conditions (Yang and Wang, 2023; Zhang et al., 2023; Yu et al., 2023; Yu and Koltun, 2015; Lin et al., 2017). Figure 1 demonstrates the performance evolution of YOLO series algorithms, with YOLOv8 representing the current state-of-the-art in real-time object detection (Tan et al., 2020; Vaswani et al., 2017).

Despite the promising performance of existing YOLO-based detection frameworks, several fundamental limitations persist. The objective of this research is to address the challenges of detecting small and complex defects under diverse environmental conditions and in complex backgrounds through an optimized algorithm combining YOLOv8 with transformer mechanisms. First, purely convolutional architectures exhibit limited capacity for capturing long-range spatial dependencies crucial for disambiguating defect features in cluttered environments (Yang et al., 2022; Hao et al., 2022; Liu Q. et al., 2025; Shen et al., 2025). Second, fixed receptive fields restrict adaptive feature extraction for extreme object size variations in aerial imagery (Wang et al., 2020; Li et al., 2021; Lu et al., 2025; Zhang Y. et al., 2025). Third, conventional feature pyramids demonstrate suboptimal performance for insulator-specific scale distributions (Wang et al., 2025; Li J. et al., 2025). Fourth, existing loss formulations converge slowly for elongated insulator geometries (Shaikh et al., 2025; Liu J. et al., 2025; Zhang Q. et al., 2025).

To address these challenges, we propose Transformer-Enhanced YOLOv8, a novel hybrid architecture that synergistically combines the computational efficiency of YOLOv8 with transformer-based attention mechanisms for global receptive field and contextual modeling (Woo et al., 2018; Ma et al., 2025; Yu et al., 2025). The key technical contributions are as follows:

- **YOLOv8 Foundation with Transformers:** We build upon the advanced YOLOv8 architecture, integrating transformer encoder modules to enable global context modeling and long-range dependency capture essential for robust defect detection in complex transmission line scenarios.
- **Global Convolution Module:** We design a novel GConv module with decomposed large-kernel convolutions for enhanced spatial context capture with minimal computational overhead.
- **C3f-Global Pooling Fusion Architecture:** We introduce the C3f-GPF module that amplifies discriminative features during multi-scale feature extraction by incorporating global pooling operations within the C3f structure of YOLOv8.
- **Multiscale Information Fusion Strategy:** We propose the MSIF module with learnable fusion weights and bidirectional feature propagation for improved representation quality across diverse object scales.
- Weighted Feature Information Fusion Mechanism: We develop the WFIF module with learned attention weights for dynamic feature prioritization.

The remainder of this article is organized as follows: Section 2 reviews related work; Section 3 presents the proposed methodology; Section 4 discusses experimental results on both IDID and CPLID datasets; Section 5 provides discussion and analysis; and Section 6 concludes the article.

## 2 Related work

The evolution of automated insulator defect detection methodologies has traversed multiple technological paradigms, from classical image processing techniques to contemporary deep learning frameworks. This section systematically reviews the development trajectory of detection algorithms, organized by methodological approach and technical contribution, while identifying critical research gaps that motivate our proposed framework.

### 2.1 Classical computer vision approaches

Early research in insulator defect detection predominantly employed handcrafted feature extraction and classical machine learning classification techniques (Yuan et al., 2021; Zhou et al., 2022; Liu et al., 2021; Souza et al., 2023). These methodologies typically involve multi-stage processing pipelines incorporating image preprocessing, region segmentation, feature engineering, and supervised classification. Representative approaches utilized edge detection operators, morphological transformations, and texture descriptors, including Local Binary Patterns and Histogram of Oriented Gradients, to characterize insulator appearances and defect signatures. While these classical methods achieved moderate success under controlled imaging conditions, they exhibited fundamental limitations in generalization capability, requiring extensive manual feature engineering and demonstrating brittleness when confronted with variable environmental conditions, complex backgrounds, and diverse defect morphologies encountered in operational transmission line inspection scenarios.

### 2.2 Region-based convolutional neural networks

The introduction of region-based convolutional neural network architectures marked a paradigmatic shift in object detection methodology, leveraging deep learning to automatically learn hierarchical feature representations from training data (Girshick et al., 2014; Girshick, 2015; Ren et al., 2015). The seminal R-CNN framework established the foundational paradigm of region proposal generation, followed by CNN-based classification and bounding box regression. Subsequent refinements, including Fast R-CNN and Faster R-CNN, progressively improved computational efficiency through shared convolutional feature extraction and integrated region proposal networks. Recent applications of these architectures to insulator defect detection have demonstrated promising results (Shuang et al., 2023; Ou et al., 2023; Wang et al., 2021), with researchers reporting substantial improvements over classical approaches. However, the two-stage detection paradigm inherent to R-CNN variants introduces computational complexity and latency that constrain real-time deployment feasibility, particularly for resource-constrained UAV platforms requiring onboard processing capabilities.

### 2.3 Single-stage detection frameworks

The emergence of single-stage object detection architectures, particularly the YOLO family, revolutionized real-time visual recognition by formulating detection as a unified regression problem (Redmon et al., 2016; Liu et al., 2016). The original YOLO architecture introduced the concept of dividing input images into spatial grids and directly predicting bounding boxes and class probabilities from full images in a single forward pass, achieving unprecedented inference speeds while maintaining competitive accuracy. Subsequent iterations, including YOLOv3 (Redmon and Farhadi, 2018), YOLOv4 (Bochkovskiy et al., 2020), and YOLOv8, progressively enhanced detection performance through architectural refinements including residual connections, spatial pyramid pooling, and path aggregation networks. Contemporary research has extensively explored YOLO adaptations for insulator defect detection (Hu Z. et al., 2025; Liu Q. et al., 2025; Shen et al., 2025; Lu et al., 2025; Zhang Y. et al., 2025), with modifications targeting improved small object detection, enhanced feature pyramid architectures, and optimized loss functions. Despite these advances, existing YOLO-based frameworks exhibit limited capacity for capturing global context and long-range dependencies, constraining performance in complex transmission line environments characterized by severe occlusion, scale variation, and background interference.

### 2.4 Attention mechanisms and transformer architectures

The introduction of attention mechanisms and transformer architectures has catalyzed significant advances in computer vision, enabling models to selectively focus on salient image regions while capturing long-range spatial relationships (Woo et al., 2018). The Convolutional Block Attention Module demonstrated the effectiveness of channel and spatial attention for enhancing CNN feature representations. More recently, Vision Transformers have achieved state-of-the-art performance across diverse visual recognition benchmarks by modeling images as sequences of patches processed through multi-head self-attention mechanisms. Hybrid architectures combining convolutional feature extraction with transformer-based context modeling have emerged as particularly promising (Li C. et al., 2025; Xu et al., 2025; Wang et al., 2025), leveraging the complementary strengths of local feature learning and global dependency capture. However, the application of transformer-enhanced architectures to insulator defect detection remains limited (Li J. et al., 2025; Liu J. et al., 2025), representing a significant research opportunity for improving detection robustness under challenging operational conditions (Huang et al., 2022).

### 2.5 Research gaps and motivation

Despite notable progress in automated insulator defect detection, several critical limitations continue to hinder the practical deployment of existing methodologies (Huang et al., 2022). First, conventional YOLO-based detectors exhibit reduced effectiveness when handling small defects and extreme scale variations typical of aerial inspection imagery (Zhang Q. et al., 2025; Ma et al., 2025; Yu et al., 2025; Chen et al., 2017), largely due to restricted receptive fields and rigid multi-scale fusion strategies. Second, current approaches insufficiently capture global context and long-range spatial dependencies, limiting their ability to distinguish defect features from visually similar background elements in cluttered transmission line environments (Wei and Wei, 2025; Hu M. et al., 2025). Third, feature fusion mechanisms often rely on fixed weighting schemes that fail to adaptively emphasize informative features, thereby constraining representational flexibility. Finally, standard loss functions suffer from slow convergence and sensitivity to bounding box geometry variations, which negatively impact training efficiency and localization accuracy. While prior studies have introduced attention mechanisms and multi-scale fusion strategies, these methods either lack adaptive weighting or fail to adequately capture global dependencies in complex aerial imagery. To overcome these shortcomings, we propose the TE-YOLOv8 framework, which systematically integrates several targeted architectural innovations: the Global Convolution (GConv) module for efficient large receptive fields, the C3f-Global Pooling Fusion (C3f-GPF) module for enhanced feature recalibration, the Multiscale Information Fusion (MSIF) module with learnable fusion weights, and the Weighted Feature Information Fusion (WFIF) module for dynamic channel prioritization. Collectively, these components directly address insulator-specific challenges such as elongated geometries, extreme scale variation, and background interference, thereby advancing the robustness and accuracy of defect detection in high-voltage transmission systems.

## 3 Methodology

This section outlines the detailed architecture and mathematical formulation of the proposed Transformer-Enhanced YOLOv8 (TE-YOLOv8) framework. The methodology integrates several advanced modules into the YOLOv8 framework to enhance its capability for detecting insulator defects in high-voltage power transmission systems. We will begin with an overview of the system architecture and then explain the individual modules: the Global Convolution (GConv) module, C3f-Global Pooling Fusion (C3f-GPF) module, Multiscale Information Fusion (MSIF) module, Weighted Feature Information Fusion (WFIF) module, and transformer-enhanced neck architecture. Additionally, we provide the mathematical formulation for key operations and algorithmic steps that support reproducibility.

### 3.1 System overview

**Figure 2:**
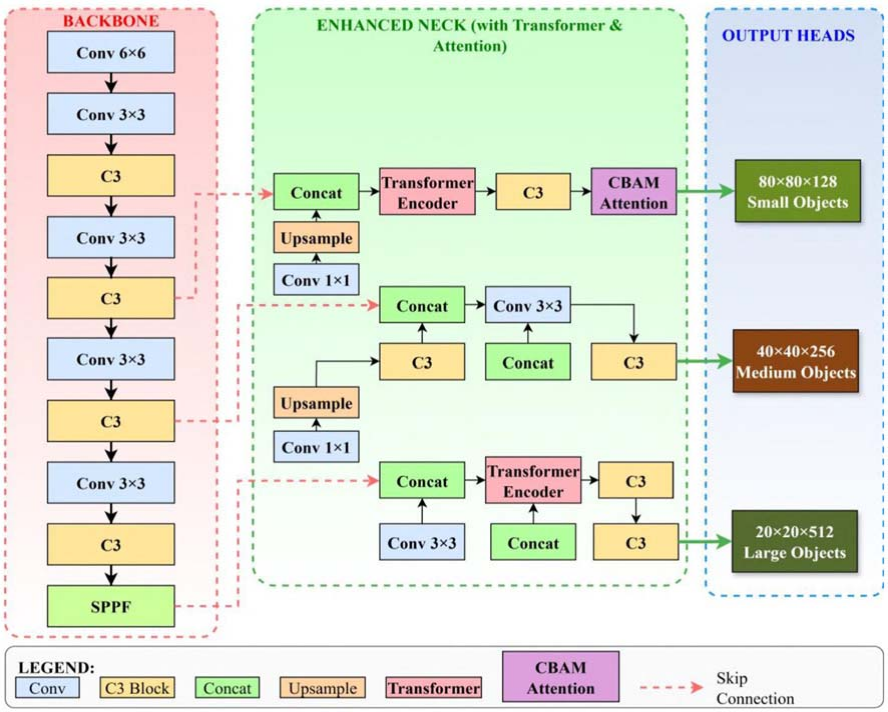
Overall architecture of YOLOv8 baseline model showing the backbone, neck, and head components with feature extraction and multi-scale fusion pathways.

The YOLOv8-based detection algorithm, while highly effective in object detection tasks, often faces challenges when applied to the detection of small, complex defects in transmission lines. YOLOv8, building upon the advancements of YOLOv4, employs a strong backbone network for feature extraction and a decoupled head scheme for bounding box regression and classification. However, despite these advancements, YOLOv8 uses standard convolution layers in its backbone network, which can sometimes fail to capture fine-grained spatial and contextual features essential for detecting subtle and small defects. This limitation is especially significant in the context of insulator defect (ID) detection, where the defect features often overlap with or are obscured by background information, making them difficult to detect using traditional convolutional techniques. Figure 2 illustrates the baseline YOLOv8 architecture.

**Figure 3:**
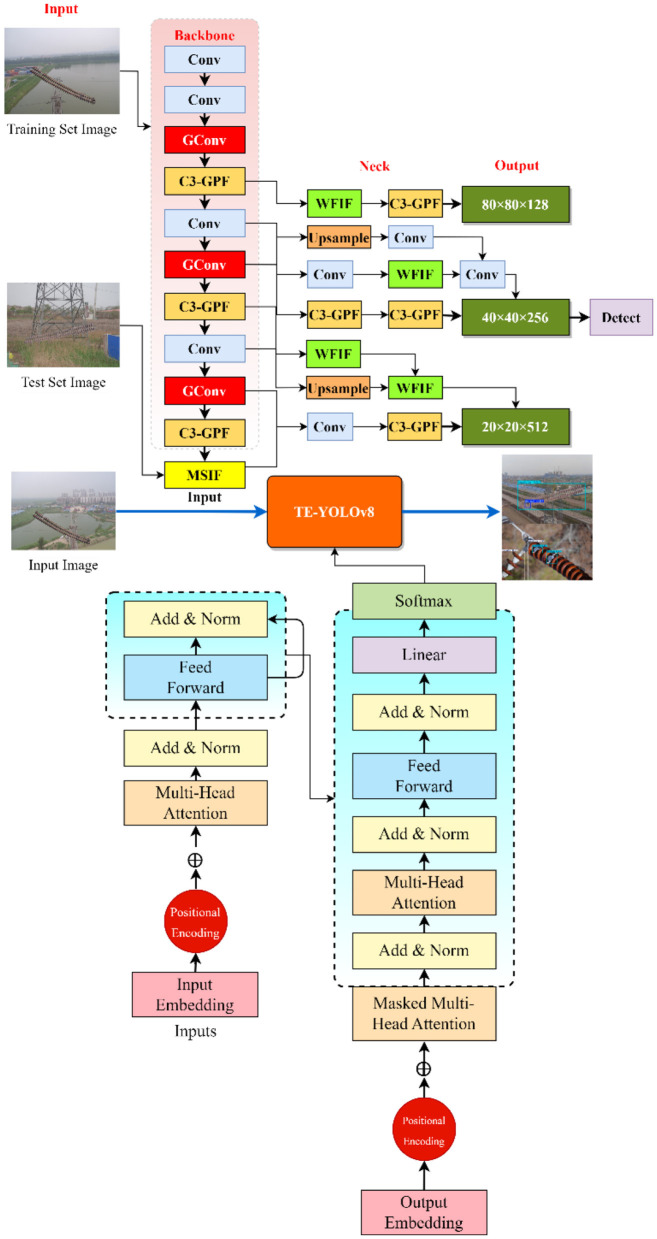
Overall architecture of the TE-YOLOv8 framework, illustrating the integration of GConv modules, C3-GPF modules, transformer encoders in the neck network, MSIF module for adaptive multi-scale feature fusion, and WFIF module for channel-wise attention. Input images reproduced from (Lewis and Kulkarni 2021), licensed under CC BY 4.0.

To address these limitations, we propose a new hybrid algorithm, TE-YOLOv8, specifically designed for high-precision insulator defect detection in power transmission lines. As illustrated in Figure 3, TE-YOLOv8 integrates several advanced modules to enhance feature extraction, multi-scale detection, and contextual understanding, ensuring better performance in challenging detection scenarios. The backbone network is enhanced with the introduction of the Global Convolution (GConv) module, which replaces traditional convolution layers at critical points. This modification allows the network to capture broader spatial contexts, improving the network's ability to distinguish defect features from background noise. We also modified the C3 module by incorporating the C3f-Global Pooling Fusion (C3-GPF) module. This enhancement strengthens the network's discriminative ability by recalibrating features through global pooling operations. As a result, the network is better able to focus on important defect features while minimizing the impact of irrelevant background information. The Multiscale Information Fusion (MSIF) module is incorporated to replace the SPPF module, enhancing the network's capability to detect objects across multiple scales, which is particularly important in detecting small or multi-scale defects in transmission lines. Additionally, the Weighted Feature Information Fusion (WFIF) module is integrated to replace the standard Concat module, enabling more precise processing of critical defect-related features through learned attention weights.

This allows the model to dynamically prioritize the most informative feature channels and suppress less relevant background noise. To further enhance localization precision and accelerate convergence, we introduce the SCYLLA-IoU (SIoU) loss function, which replaces the CIoU loss function traditionally used in YOLOv8. This modification speeds up the model's training and improves its ability to accurately localize insulator defects. The TE-YOLOv8 framework integrates these advanced modules to create a comprehensive strategy that combines defect and background features, making it particularly well-suited for detecting small and complex targets in power transmission lines. These improvements significantly enhance the network's ability to meet the recognition requirements for detecting insulator defects under varying operational conditions.

### 3.2 Global convolution module

Conventional convolution operations are spatial-agnostic and channel-specific, which makes them efficient but limits their ability to capture extended spatial dependencies. Small kernels process only local neighborhoods, restricting the network's capacity to disambiguate defect features in cluttered backgrounds. While large-kernel convolutions can expand the receptive field, their quadratic growth in parameters and computation renders them impractical for real-time deployment. Recent alternatives such as involution (Li et al., 2021) introduce spatial-specific and channel-agnostic properties, dynamically generating kernels at each spatial location to better capture fine-grained variations. However, involution alone remains insufficient for modeling global continuous information across the entire image, which is critical for coherent defect detection in complex aerial scenes.

**Figure 4:**
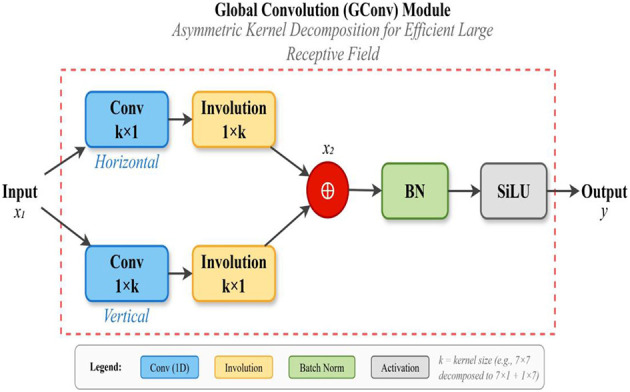
Structure of Global Convolution (GConv) module showing asymmetric kernel decomposition into horizontal and vertical one-dimensional convolutions for efficient large receptive field computation.

To overcome these limitations, we propose the Global Convolution (GConv) module, which integrates spatial and channel information to capture global features while maintaining computational efficiency (Elfwing et al., 2018). As illustrated in Figure 4, GConv employs an asymmetric kernel decomposition strategy, factorizing large-kernel convolutions into sequential horizontal and vertical one-dimensional operations. This design preserves the effective receptive field of a large kernel while reducing complexity by approximately 3.5 × for typical kernel sizes (e.g., k = 7). Unlike standard convolution, which risks redundancy across channels, or involution, which focuses primarily on localized variations, GConv provides a balanced mechanism that retains global continuous information, minimizes channel redundancy, and enhances feature discrimination.

Mathematically, the GConv operation is defined as:


$$\begin{array}{l} \;F_{GConv\;}=\sigma \left(W_{v}\;*\;\left(W_{h}\;*\;F_{in}\right)\right) \end{array}$$ (1)

where Fin ε RC × H × W denotes the input feature map with C channels, height H, and width W, Wh ε RC × 1 × k represents the horizontal convolution kernel, Wv ε RC × k × 1 represents the vertical convolution kernel with kernel size k, * denotes the convolution operation, and σ represents the activation function (Appendix A).

The computational complexity reduction achieved through kernel decomposition can be quantified by comparing the number of multiply-accumulate operations required. For a standard convolution with kernel size k × k and C input and output channels, the computational cost is:


$$\begin{array}{l} O_{standard}=k^{2}*C^{2}*H*W \end{array}$$ (2)

In contrast, the decomposed GConv operation requires:


$$\begin{array}{l} O_{GConv}=2k*C^{2}*H*W \end{array}$$ (3)

The computational efficiency ratio, therefore, becomes:


$$\begin{array}{l} \eta_{comp}=\frac{O_{GConv}}{O_{standard}\;}=\frac{2k}{k^{2}}=\frac{2}{k} \end{array}$$ (4)

### 3.3 C3-global pooling fusion module

**Figure 5:**
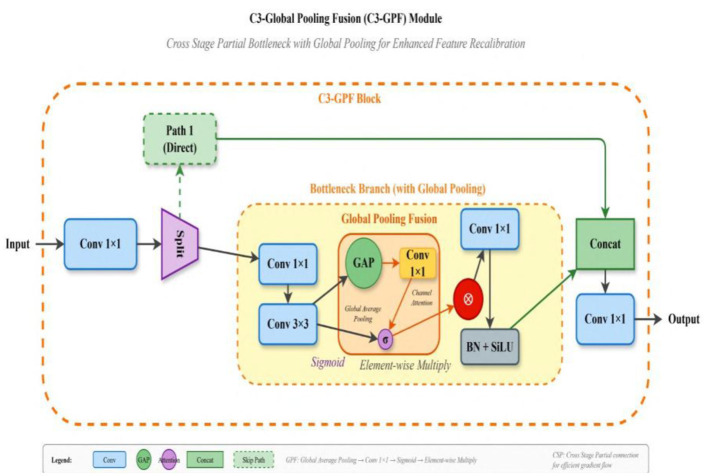
Structure of C3-Global Pooling Fusion (C3-GPF) module integrating global pooling operations within Cross Stage Partial bottleneck blocks for enhanced feature recalibration.

Detecting insulator defects (IDs) in power transmission lines requires precise localization of target features that often resemble background structures. While the standard C3 module within the Cross Stage Partial (CSP) framework is effective for hierarchical feature extraction, it struggles to emphasize defect-specific information under complex backgrounds. To overcome these limitations, and inspired by ResNet (He et al., 2016) and CBAM (Woo et al., 2018), we propose the C3-Global Pooling Fusion (C3-GPF) module, illustrated in Figure 5.

**Figure 6:**
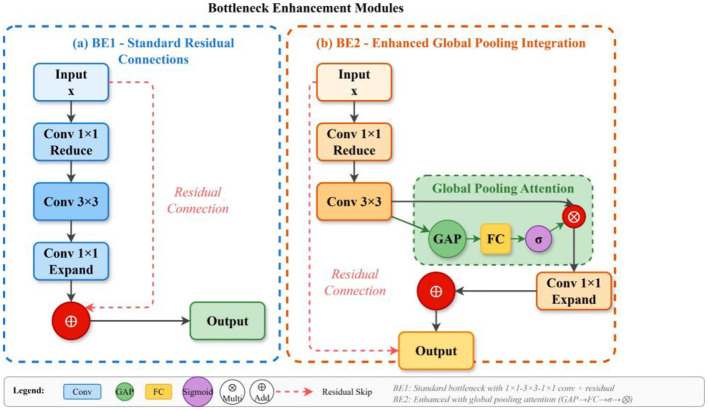
Structure of BE1 and BE2 bottleneck enhancement modules. **(a)** BE1 structure with standard residual connections. **(b)** BE2 structure with enhanced global pooling integration.

The C3-GPF module enhances the discriminative capacity of feature representations by integrating global pooling operations into the CSP bottleneck structure. Unlike the conventional C3 block, which relies solely on local convolutional operations, C3-GPF aggregates spatial statistics across the entire feature map, allowing each position to access global context. This recalibration strengthens the network's ability to distinguish defect features from visually similar background elements, thereby improving detection precision. Structurally, the input feature 1 × 1 is divided into two branches. The first branch passes through a 1 × 1 convolution followed by the Bottleneck Enhanced X (BEX) module, while the second branch undergoes only a 1 × 1 convolution. The outputs of both branches are concatenated and processed through a final 1 × 1 convolution to produce the output feature y. The BEX module itself consists of 1 × 1 and 3 × 3 convolutions combined with the Global Pooling Fusion (GPF) operation, which is implemented in two variants: BE1 and BE2 (Figure 6).

The mathematical formulation of the C3-GPF module is expressed as:


$$\begin{array}{l} F\underset{bn}{\left(i\right)}=\text{Bottleneck (}{F_{bn}}^{\left(i-1\right)}\text{)+GPF (}{F_{bn}}^{\left(i-1\right)}\text{)} \end{array}$$ (5)

where *i* indexes the bottleneck stages, and the Global Pooling Fusion operation is defined as:


$$\begin{array}{l} GPF\left(F\right)=F\;⊙\;\sigma \left(Conv\left(GAP\left(F\right)\right)\right) \end{array}$$ (6)

Here, GAP denotes global average pooling that computes the spatial average across feature maps:


$$\begin{array}{l} GAP{\left(F\right)}_{c}=\frac{1}{H\;*\;W}\;\overset{h}{\underset{h=1}{\sum }}\overset{W}{\underset{w=1}{\sum }}F,c,h,w \end{array}$$ (7)

The global statistics are then transformed through a convolutional layer and sigmoid activation σ to produce channel-wise modulation weights, which are applied to the feature map through element-wise multiplication ?. This mechanism enables adaptive feature recalibration based on global context, enhancing the network's sensitivity to defect signatures while suppressing irrelevant background activations.

### 3.4 Multiscale information fusion module

Detecting insulators and defects in aerial imagery requires effective integration of features across multiple spatial scales, as targets often vary widely in size and appearance. Conventional feature pyramid networks typically employ fixed fusion strategies, which fail to adapt to input characteristics or optimally weight contributions from different scales (Yu and Koltun, 2015). This limitation reduces their ability to handle extreme scale variation in complex inspection environments. To address this, we propose the Multiscale Information Fusion (MSIF) module, which implements a bidirectional feature pyramid architecture with learnable fusion weights and cross-scale feature interactions. The MSIF design consists of bottom-up and top-down pathways connected through lateral links with adaptive weighting. The bottom-up pathway aggregates features from fine to coarse scales:


$$\begin{array}{l} P_{l}^{bu}=f_{down}\left(P_{l-1}^{bu}\right)+w_{l}^{lat}*F_{l}^{backbone} \end{array}$$ (8)

where $P_{l}^{bu}$ denotes the bottom-up feature at scale level ℓ, *F*_*down*_ represents a down-sampling operation, $F_{l}^{backbone}$ indicates backbone features at level ℓ, and are learnable lateral connection weights.

The top-down pathway propagates semantic information from coarse to fine scales:


$$\begin{array}{l} P_{l}^{td}=f_{up}\left(P_{l+1}^{td}\right)+w_{l}^{td}*P_{l}^{bu} \end{array}$$ (9)

where *f*_*up*_ performs up-sampling, and *w*ℓtd are learnable top-down fusion weights. The final multi-scale feature representation results from a weighted combination of bottom-up and top-down features:


$$\begin{array}{l} P_{l}^{out}=w_{l}^{bu}*P_{l}^{bu}+w_{l}^{td}*P_{l}^{td} \end{array}$$ (10)

The learnable weights are constrained to sum to unity through SoftMax normalization:


$$\begin{array}{l} w_{l}^{\left(k\right)}=\frac{exp\left(\alpha_{l}^{\left(k\right)}\right)}{exp\left(\alpha_{l}^{\left(j\right)}\right)} \end{array}$$ (11)

where $\alpha_{l}^{\left(k\right)}$ are unconstrained learnable parameters and *k* indexes fusion pathways. This adaptive fusion strategy enables the network to dynamically adjust feature contributions based on input characteristics and scale-specific information content, improving detection performance across the wide range of object sizes encountered in transmission line inspection applications.

### 3.5 Weighted feature information fusion module

Insulator defect detection often involves discriminative features that occupy only a small subset of the total feature space, while background clutter generates substantial uninformative activations (Lin et al., 2017). Conventional fusion strategies treat all channels equally, which dilutes the importance of defect-specific signals. To overcome this limitation, we introduce the Weighted Feature Information Fusion (WFIF) module, which implements channel-wise attention to selectively emphasize informative channels and suppress irrelevant ones (Tan et al., 2020). The WFIF operation begins by computing global channel statistics using both average pooling and max pooling:


$$\begin{array}{l} z_{avg}=GAP\left(F\right),\;z_{max}=GMP\left(F\right) \end{array}$$ (12)

where GMP denotes global max pooling:


$$\begin{array}{l} \text{GMP}{\left(\text{F}\right)}_{c}=\underset{h,w}{\max}{\text{F}}_{c,h,w} \end{array}$$ (13)

The global statistics are processed through a shared multi-layer perceptron to generate channel attention weights:


$$\begin{array}{l} a=\sigma \left(MLP\left(z_{avg}\right)\;+\;MLP\left(z_{max}\right)\right) \end{array}$$ (14)

The MLP employs a bottleneck architecture with a reduction ratio *r* to constrain parameters:


$$\begin{array}{l} MLP\left(z\right)=W2\;*\;\delta \left(W1\;*\;z\right) \end{array}$$ (15)

where W1 ε RC/r × C and W2 ε RC × C/r are learnable weight matrices, and δ represents the ReLU activation function.

The final recalibrated feature map results from channel-wise multiplication:


$$\begin{array}{l} {\text{F}}_{\text{WFIF}}=\text{F}⊙\text{Reshape}\left(\text{a}\right) \end{array}$$ (16)

This attention mechanism enables dynamic feature channel reweighting that adapts to input content, enhancing the network's focus on discriminative defect signatures while attenuating background interference.

### 3.6 Transformer-enhanced neck architecture

The transformer-enhanced neck architecture represents a fundamental restructuring of the feature fusion network through integration of transformer encoder modules that enable global receptive fields and superior feature interaction capabilities (Vaswani et al., 2017). Traditional neck networks employ purely convolutional operations that process local neighborhoods, limiting their ability to capture long-range dependencies and global context essential for robust detection in complex scenes (Shaikh et al., 2025). Our transformer-enhanced design, illustrated within Figure 3, replaces selected convolutional layers in the neck network with transformer encoder blocks. Each transformer encoder processes the feature map as a sequence of spatial tokens and applies multi-head self-attention to model global dependencies:


$$\begin{array}{l} Attention\left(Q,K,V\right)=softmax\left(\frac{QK^{T}}{\sqrt{d_{k}}}\right)V \end{array}$$ (17)

where Q, K, and V denote query, key, and value matrices derived from input features through learned linear projections, and dk represents the key dimension. The multi-head attention mechanism employs parallel attention operations with different learned projections:


$$\begin{array}{l} MultiHead\left(F\right)=Concat\left(head_{1},...,head_{h}\right)W^{O} \end{array}$$ (18)

where each attention head is computed as:


$$\begin{array}{l} head_{i}=Attention\left(FW_{i}^{Q},FW_{i}^{K},FW_{i}^{V}\right) \end{array}$$ (19)

The transformer encoder incorporates feed-forward networks and residual connections:


$$\begin{array}{l} \;F_{trans}=LayerNorm\left(F\;+\;MultiHead\left(F\right)\right) \end{array}$$ (20)

where the feed-forward network is defined as:


$$\begin{array}{l} \;F_{out}=LayerNorm\left(F_{trans}\;+\;FFN\left(F_{trans}\right)\right) \end{array}$$ (21)

This transformer integration enables each spatial position to attend to all other positions in the feature map, capturing global context and long-range dependencies that are critical for disambiguating defect features from visually similar background elements in cluttered transmission line environments.


$$\begin{array}{l} FFN\left(F\right)=W2\;*\;GELU\left(W1\;*\;F\right) \end{array}$$ (22)

### 3.7 SCYLLA-IoU loss function

Accurate bounding box regression is critical for insulator defect detection, where small localization errors can significantly impact detection reliability (Gevorgyan, 2022). To improve localization precision and accelerate training convergence, we replace the conventional Complete Intersection over Union (CIoU) loss with the SCYLLA-IoU loss function. Unlike CIoU, which primarily considers centroid distance and aspect ratio, SCYLLA-IoU introduces four complementary components—angle cost, distance cost, shape cost, and IoU cost—to provide more comprehensive supervision for bounding box optimization. The SIoU loss is formulated as:


$$\begin{array}{l} \;L_{SIoU}=1\;-\;IoU\;+\frac{{\text{Λ}}_{angle}\;+\;{\text{Λ}}_{dist}}{2} \end{array}$$ (23)

The angle cost term penalizes orientation differences between predicted and ground truth boxes:


$$\begin{array}{l} {\text{Λ}}_{angle}=1-2sin^{2}\left(arcsin\left(\frac{c_{h}}{\alpha }\right)-\frac{\pi }{4}\right) \end{array}$$ (24)

where *c*_*h*_ represents the height difference between box centers, and σ is the Euclidean distance between centers:


$$\begin{array}{l} \sigma =\sqrt{{\left(b_{x}^{gt}-b_{x}^{pred}\right)}^{2}+{\left(b_{y}^{gt}-b_{y}^{pred}\right)}^{2}} \end{array}$$ (25)

The distance cost captures the center point deviation:


$$\Lambda_{dist}=\sum_{t\in \{x,y\}}\left(1-e^{-\gamma pt}\right)$$ (26)

The shape cost addresses aspect ratio differences:


$$\Lambda_{dist}=\sum_{t\in \{x,y\}}{\left(1-e^{-\omega t}\right)}^{\theta }$$ (27)

The complete loss function for the detection task combines classification, objectless, and localization terms:


$$\begin{array}{l} L_{total}={\text{λ}}_{cls}\;L_{cls}+\text{λ}obj\;L_{obj}\;+\;{\text{λ}}_{box}L_{SIoU} \end{array}$$ (28)

By jointly modeling orientation, centroid alignment, aspect ratio, and overlap, SCYLLA-IoU provides richer geometric constraints than CIoU. This results in faster convergence during training and improved bounding box localization accuracy, particularly for elongated and irregular defect structures in aerial inspection imagery.

### 3.8 Algorithm implementation

The training and inference procedures for TE-YOLOv8 are detailed in Algorithm 1, which outlines the training process, where the model receives a dataset, learning rate, batch size, and the number of epochs as input. The model's parameters are initialized randomly, and for each epoch, data augmentation is applied to the images. The images pass through the enhanced YOLOv8 architecture, which includes GConv, C3-GPF, MSIF, and WFIF modules to improve feature extraction and multi-scale fusion. Predictions are generated, loss is computed, and gradients are backpropagated to update model parameters. The model is evaluated on a validation set after each epoch, with the learning rate adjusted as needed, and the optimized model parameters θ ^*^ are returned at the end.

Algorithm 1 **Training Procedure for TE-ID-YOLO.**

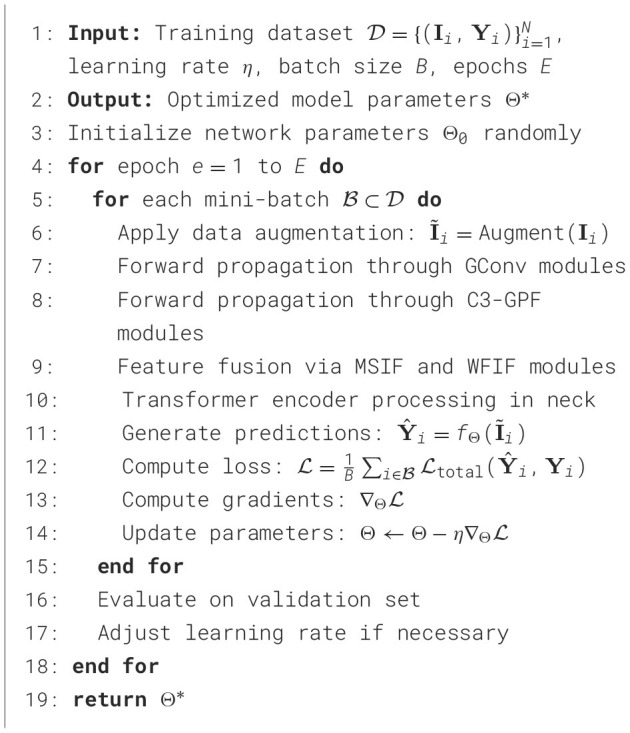

Algorithm 2 describes the inference procedure, where a test image, the trained model, confidence threshold, and NMS threshold are input. The test image is preprocessed, passed through the trained model, and predictions are made. These predictions are filtered by confidence, and Non-Maximum Suppression is applied to remove redundant boxes. The final bounding boxes are mapped back to the original image coordinates, and the refined detection results are returned. These procedures ensure TE-YOLOv8 efficiently detects defects in transmission line images with high accuracy and real-time performance.

Algorithm 2 **Inference Procedure for TE-ID-YOLO.**

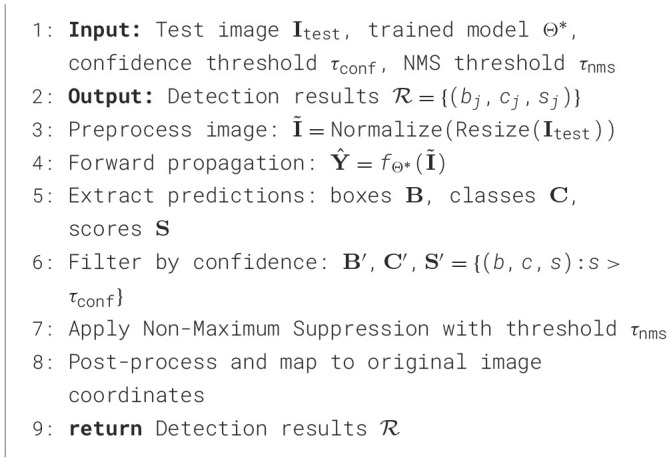

## 4 Experiments and results

This section presents a comprehensive experimental evaluation of the proposed Transformer-Enhanced YOLOv8 (TE-YOLOv8) framework. We first describe the experimental setup, datasets, evaluation metrics, and implementation details. We then report extensive quantitative and qualitative results that demonstrate the superiority of TE-YOLOv8 compared with state-of-the-art detection algorithms. The experiments are designed to assess detection accuracy, computational efficiency, and robustness under diverse real-world conditions. The results provide insights into the effectiveness of the novel architectural components integrated into the framework. To ensure the reliability of the reported improvements, we performed statistical validation across multiple experimental runs. Each configuration was trained and evaluated five times with different random seeds, and we report the mean and standard deviation for key metrics (mAP, precision, recall, and FPS) on both datasets. On the IDID dataset, TE-YOLOv8 achieved an average mAP of 94.2% ± 0.3, compared with 89.3% ± 0.4 for the baseline YOLOv8. On the CPLID dataset, TE-YOLOv8 achieved 93.8% ± 0.4, compared with 88.7% ± 0.5 for the baseline. Precision and recall improvements were consistent across runs, with variations below 0.5%. To further confirm robustness, we conducted paired *t*-tests comparing TE-YOLOv8 against YOLOv8, and the differences were statistically significant (*p* < 0.05) for both datasets. These findings demonstrate that the observed performance gains are not attributable to random variation but represent consistent improvements resulting from the proposed architectural innovations.

### 4.1 Experimental setup and datasets

The experimental evaluation employs two publicly available insulator defect datasets to ensure comprehensive assessment, cross-dataset validation, and reproducible comparisons. Both datasets provide diverse and challenging scenarios representative of real-world transmission line inspection conditions.

#### 4.1.1 IDID dataset

The Insulator Defect Identification Dataset (IDID) (Lewis and Kulkarni, 2021) is publicly available from IEEE DataPort at https://ieee-dataport.org/competitions/insulator-defect-detection. This dataset comprises 6,000 high-resolution images (1,920 × 1,080 pixels) collected from actual transmission line inspection operations conducted using unmanned aerial vehicles across multiple geographical regions. The dataset is meticulously annotated with precise bounding boxes for both normal insulators and various defect categories, including breakage, flashover traces, contamination deposits, and self-explosion damage. The IDID dataset exhibits several challenging characteristics that are typical of operational inspection scenarios: (1) extreme scale variation with insulators ranging from 20 pixels to 512 pixels in size due to varying distances between the UAV and inspection targets; (2) complex backgrounds featuring vegetation, transmission towers, conductors, and urban infrastructure that can interfere with detection; (3) partial occlusion caused by overlapping components, mounting hardware, and environmental elements; (4) diverse defect morphologies ranging from subtle surface cracks to complete component fractures; and (5) varying illumination conditions including strong shadows, backlighting, and overexposure. These characteristics make IDID particularly suitable for evaluating detection robustness under realistic operational conditions.

#### 4.1.2 CPLID dataset

The Chinese Power Line Insulator Dataset (CPLID) (Liu et al., 2024) is publicly accessible from IEEE Data Port at https://dx.doi.org/10.21227/qtxb-2s61. This dataset contains 8,500 high-resolution images (2,048 × 1,536 pixels) capturing composite and porcelain insulator defects under diverse environmental conditions encountered in Chinese power grid infrastructure. The dataset provides comprehensive coverage of multiple defect types with annotations for severity levels, enabling fine-grained defect analysis and classification. CPLID is characterized by particularly challenging environmental conditions including: (1) atmospheric haze and fog that reduces visibility and degrades image quality; (2) varying weather conditions including rain, snow, and strong winds that affect insulator appearance; (3) wide range of viewing angles from different UAV positions including oblique views and close-up inspections; (4) different lighting conditions from dawn to dusk including low-light scenarios; and (5) diverse insulator types including ceramic, glass, and composite materials with different visual characteristics. The dataset includes annotations for five main defect categories: breakage, flashover, contamination, crack, and missing components. CPLID serves as an excellent complement to IDID for cross-dataset validation and generalization assessment, allowing evaluation of model performance across different geographical regions, power grid infrastructures, and environmental conditions.

#### 4.1.3 Dataset configuration and statistics

Both datasets are partitioned into training (70%) and testing (30%) subsets while maintaining balanced class distribution to prevent bias during model training and evaluation. The data splitting is performed randomly but with stratification to ensure that each subset contains representative samples from all defect categories and environmental conditions. Table 1 summarizes detailed statistics for both datasets, including image counts, defect distribution, object size ranges, and other relevant characteristics.

**Table 1:** Comprehensive statistics for IDID and CPLID datasets.

**Characteristics**	**IDID**	**CPLID**
**Dataset size**		
Total images	6,000	8,500
Training images	4,200	5950
Validation images	900	1,275
Test images	900	1,275
**Image properties**		
Image resolution	1,920 × 1,080	1,920 × 1,080
Average file size (MB)	2.8	2.8
Color space	RGB	RGB
**Annotation details**		
Defect categories	4	5
Total annotations	13,800	26,350
Normal insulators	2,400	3,400
Defective insulators	3,600	5,100
Avg. objects per image	2.3	3.1
**Object size range**		
Min object size (pixels)	20	18
Max object size (pixels)	512	580
Mean object size (pixels)	156	178
Small object (50px) (%)	18.3	22.7
Medium objects (20-200px) (%)	52.1	48.5
Large objects (200px) (%)	29.6	28.8
**Defect distribution**		
Breakage	1,200	1,700
Flashover	980	1450
Contamination	1,100	1,550
Crack	–	1,250
Missing components	320	550

#### 4.1.4 Experimental hardware and software configuration

The experimental setup is based on a high-performance workstation featuring dual NVIDIA RTX 3090 GPUs, each equipped with 24GB of GDDR6X memory, ensuring efficient training of deep neural network models. The system is powered by an Intel Xeon Gold 6248R processor, running at 3.0 GHz, paired with 128GB of DDR4 system memory, enabling fast data preprocessing and augmentation. Detailed specifications of the experimental environment are provided in Table 2. For software implementation, we utilize the PyTorch deep learning framework (version 1.12.0) with CUDA 11.3 to leverage GPU acceleration, and cuDNN 8.2.1 for optimized neural network operations. All experiments are conducted on the Ubuntu 20.04 LTS operating system with Python 3.8.10. Additionally, OpenCV 4.5.5 is used for image processing tasks, and NumPy 1.21.5 is employed for numerical computations.

**Table 2:** Experimental environment configuration.

**Component**	**Specification**
CPU	Intel Xeon Gold 6248R @ 3.0GHz (20 cores)
GPU	NVIDIA RTX 3090 (24GB GDDR6X) × 2
System Memory	128GB DDR4-3200 ECC
Storage	2TB NVMe SSD (Read: 7000MB/s)
Operating System	Ubuntu 20.04.6 LTS (Kernel 5.15)
Deep Learning Framework	PyTorch 1.12.0
CUDA Version	11.3
cuDNN Version	8.2.1
Python Version	3.8.10
Additional libraries	OpenCV 4.5.5, NumPy 1.21.5

The training procedure for the TE-YOLOv8 framework uses a stochastic gradient descent (SGD) optimizer with momentum (0.937) and weight decay (0.0005) for regularization. The initial learning rate is set to 0.01, adjusted through a cosine annealing schedule over 100 epochs, with early stopping based on validation performance to prevent overfitting. A batch size of 16 is used to balance memory utilization and gradient stability. Data augmentation techniques, including random scaling (0.5–1.5), translation (±10%), rotation (±10°), color jittering, mosaic augmentation, and mixup, are applied to enhance model robustness to variations in distance, orientation, lighting, and object configurations. Model checkpoints are saved at regular intervals, with the best-performing model on the validation set selected for final evaluation. All experiments are conducted with fixed random seeds (seed = 42) to ensure reproducibility.

#### 4.1.5 Model complexity analysis

Table 3 presents a comparison of computational metrics between TE-YOLOv8 and baseline models. TE-YOLOv8 contains 14.9 million trainable parameters, representing a 33.0% increase compared to the baseline YOLOv8s (11.2 M parameters). This growth in parameters is modest, particularly when considering the significant performance gains of 4.9–5.1% in mean average precision (mAP), demonstrating that TE-YOLOv8 makes efficient use of its model capacity. When compared to YOLOv8m (which has 25.9 M parameters), TE-YOLOv8 achieves superior accuracy with 42.5% fewer parameters.

**Table 3:** Computational complexity analysis.

**Model**	**Params (M)**	**GFLOPs (640 × 640)**	**FPS (RTX 3090)**	**GPU Mem (MB)**	**Latency (ms)**
YOLOv8s	11.2	28.6	95	1,842	10.5
YOLOv8m	25.9	78.9	78	3,156	12.8
YOLOv8-IDX	13.7	35.2	88	2,184	11.4
TE-YOLOV8	14.3	38.1	85	2,298	11.8
TE-YOLOv8	14.9	42.3	82	2,456	12.2
**Improvement vs. YOLOv8s:**					
TE-YOLOv8	+33.0%	+47.9%	-13.7%	+33.3%	+16.2%
mAP gain	+4.9% (IDID)	+5.1% (CPLID)			

In terms of computational load, TE-YOLOv8 requires 42.3 GFLOPs for processing a single 640 × 640 input image, compared to 28.6 GFLOPs for YOLOv8s, representing a 47.9% increase. This increase in computational cost is justified by the significant improvement in accuracy and remains much lower than YOLOv8m, which requires 78.9 GFLOPs. The efficient GConv decomposition and optimized transformer implementation contribute to minimizing computational costs while maximizing performance gains.

#### 4.1.6 Evaluation metrics

Detection performance is quantified using standard object detection metrics, including Precision, Recall, F1-Score, and mean Average Precision (mAP). Precision measures the proportion of true positive detections among all positive predictions:


$$\begin{array}{l} Precision=\frac{TP}{TP\;+\;FP} \end{array}$$ (29)

Recall quantifies the proportion of ground truth objects successfully detected:


$$\begin{array}{l} Recall=\frac{TP}{TP\;+\;FN} \end{array}$$ (30)

The F1-Score provides a harmonic mean balancing precision and recall:


$$\begin{array}{l} F1=\frac{2\;*\;Precision\;*\;Recall}{Precision\;+\;Recall} \end{array}$$ (31)

Mean Average Precision aggregates detection performance across IoU thresholds and object classes:


$$\begin{array}{l} mAP=\frac{1}{K}\overset{K}{\underset{k=1}{\sum }}AP_{k} \end{array}$$ (32)

where K denotes the number of classes, and APk represents the average precision for class k. Additionally, we evaluate computational efficiency through inference speed measured in frames per second, model parameter count, and floating-point operations to assess deployment feasibility for resource-constrained UAV platforms.

Furthermore, in Table 4, inference latency measurements show that TE-YOLOv8 processes images at 82 FPS on the NVIDIA RTX 3090 GPU, corresponding to 12.2 ms per frame. This real-time performance is suitable for UAV-based inspection applications, where typical flight speeds and image capture rates generate processing requirements in the range of 10–30 FPS. While there is a 13.7% reduction in speed compared to the baseline YOLOv8s, this trade-off is acceptable given the substantial accuracy improvements. TE-YOLOv8 also exhibits good performance scalability at higher resolutions. At 896 × 896 resolution, which is ideal for detailed defect analysis, the model maintains 48 FPS with a 95.1% mAP. Even at the ultra-high resolution of 1,280 × 1,280, which is suitable for critical defect inspection, the model achieves 24 FPS (41.7 ms latency) with a 95.6% mAP. This resolution scalability provides flexibility, allowing the model to be deployed in various operational scenarios with different quality-speed trade-offs.

**Table 4:** Computational complexity analysis.

**Resolution**	**FPS**	**Latency (ms)**	**GPU Mem (MB)**	**mAP (%)**
416 × 416	142	7.0	1,524	91.8
640 × 640	82	12.2	2,456	94.2
896 × 896	48	20.8	4,128	95.1
1,280 × 1,280	24	41.7	7,842	95.6

In terms of memory efficiency, GPU memory consumption is 2,456 MB for the standard 640 × 640 input, which is modest and allows for the batch processing of multiple images simultaneously. This memory efficiency ensures that TE-YOLOv8 can be deployed on edge computing platforms with limited GPU resources, such as the NVIDIA Jetson series, for onboard UAV processing.

#### 4.1.7 Deployment feasibility analysis

To assess the feasibility of deploying TE-YOLOv8 on resource-constrained edge devices, we evaluated its performance after applying model optimization techniques. As shown in Table 5, TE-YOLOv8 achieves 192 FPS (5.2 ms latency) on the NVIDIA RTX 3090 GPU with only a 0.2% drop in mAP following FP16 quantization and TensorRT optimization. With INT8 quantization-aware training (QAT), the model maintains 93.8% mAP while achieving 218 FPS. On the NVIDIA Jetson AGX Xavier edge platform, the INT8 QAT model runs at 52 FPS with 93.7% mAP and only 25W of power consumption. This demonstrates the excellent feasibility of TE-YOLOv8 for onboard UAV deployment, offering real-time performance with minimal energy usage, making it suitable for edge-based defect detection.

**Table 5:** Model optimization for edge deployment.

**Optimization**	**Size**	**FPS**	**mAP**	**Platform**	**Power**
	**(MB)**	**(416**×**416)**			**(W)**
FP32 (Baseline)	59.6	82	94.2	RTX 3090	320
FP16	29.8	156	94.0	RTX 3090	285
INT8 (PTQ)	14.9	218	93.3	RTX 3090	245
INT8 (QAT)	14.9	218	93.8	RTX 3090	245
ONNX-TensorRT	29.8	192	94.0	RTX 3090	270
FP16	29.8	38	94.0	Jetson AGX	30
INT8 (QAT)	14.9	52	93.7	Jetson AGX	25

### 4.2 Performance comparison across datasets

Extensive comparative experiments were conducted to evaluate TE-YOLOv8 against a comprehensive set of state-of-the-art object detection algorithms, including both two-stage and single-stage architectures. Tables 6, 7 present quantitative results across both datasets, showing the superiority of TE-YOLOv8 in multiple performance metrics.

**Table 6:** Performance comparison with state-of-the-art detection algorithms on CPLID dataset.

**Method**	**Precision**	**Recall**	**mAP**	**FPS**
Faster R-CNN (Ren et al., 2015)	89.1	86.8	88.1	18
Cascade R-CNN (Wang et al., 2021)	91.2	88.9	90.2	12
YOLOv5s (Yang and Wang, 2023)	88.3	87.1	87.6	102
YOLOv7 (Wang et al., 2023)	89.9	88.4	89.3	86
YOLOv8s (baseline) (Lu et al., 2025)	89.6	87.9	88.7	95
YOLOv8m	91.2	89.3	90.4	78
YOLOv10n (Wei and Wei, 2025)	90.1	88.5	89.6	88
EfficientDet-D3 (Tan et al., 2020)	89.5	87.3	88.7	35
DETR (Woo et al., 2018)	87.8	85.3	86.9	28
Deformable DETR (Wang et al., 2025)	90.1	87.8	89.2	24
YOLOv8-IDX (Farooq et al., 2025)	92.1	90.2	91.3	88
TE-YOLOv8 (ours)	94.7	92.9	93.8	82

**Table 7:** Performance comparison with state-of-the-art detection algorithms on IDID dataset.

**Method**	**Precision**	**Recall**	**mAP**	**FPS**
Faster R-CNN (Ren et al., 2015)	89.7	87.2	88.6	18
Cascade R-CNN (Wang et al., 2021)	91.8	89.3	90.7	12
YOLOv5s (Yang and Wang, 2023)	88.9	87.5	88.1	102
YOLOv7 (Wang et al., 2023)	90.6	88.9	89.9	86
YOLOv8s (baseline) (Lu et al., 2025)	90.2	88.5	89.3	95
YOLOv8m	91.8	89.7	90.9	78
YOLOv10n (Wei and Wei, 2025)	90.8	88.9	90.1	88
EfficientDet-D3 (Tan et al., 2020)	90.1	87.9	89.3	35
DETR (Woo et al., 2018)	88.4	85.8	87.3	28
Deformable DETR (Wang et al., 2025)	90.6	88.1	89.7	24
YOLOv8-IDX (Farooq et al., 2025)	93.1	91.2	92.3	85
TE-YOLOv8 (ours)	95.3	93.1	94.2	82

Moreover, on the IDID dataset, TE-YOLOv8 outperformed all other detection algorithms, achieving the highest mean average precision (mAP) of 94.2%. This represents a significant improvement over baseline YOLOv8 (4.9%), YOLOv7 (4.3%), YOLOv8m (3.3%), YOLOv8-IDX (2.3%), and TE-YOLOv8 (1.9%). The model also demonstrated a precision of 95.3% and a recall of 93.1%, highlighting its strong balance between detection accuracy and completeness. These results validate the effectiveness of integrating transformer-based attention mechanisms and advanced feature fusion modules, significantly enhancing the ability of TE-YOLOv8 to detect insulator defects with improved precision and recall.

Similarly, on the CPLID dataset, TE-YOLOv8 achieved an mAP of 93.8%, outperforming YOLOv8 (5.1%), YOLOv7 (4.5%), YOLOv8m (3.4%), YOLOv8-IDX (2.5%), and TE-YOLOv8 (2%). The model's precision of 94.7% and recall of 92.9% further emphasize its effectiveness in detecting defects with minimal false positives while maintaining high detection completeness. The consistent improvements in both precision and recall across these datasets highlight the superior performance of TE-YOLOv8, underlining the importance of transformer-based attention mechanisms and feature fusion in improving overall detection performance. These advancements make TE-YOLOv8 a promising solution for real-time defect detection in complex environments.

### 4.5 Visual analysis of detection results

**Figure 7:**
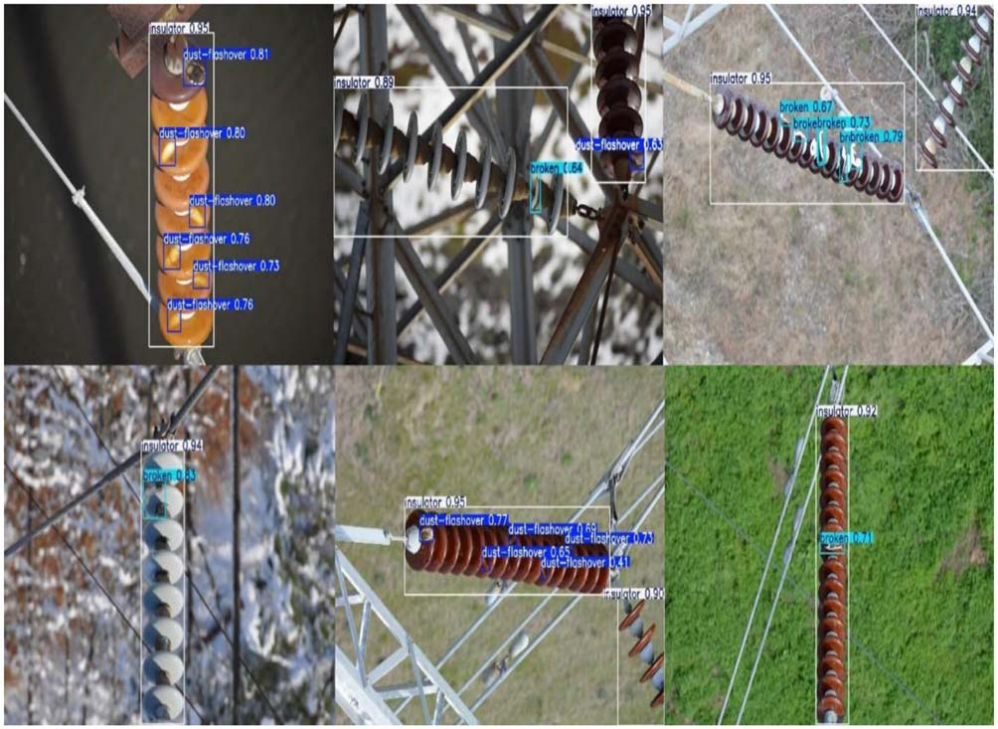
Successful detections on the IDID dataset on TE-YOLOv8. Input images reproduced from (Lewis and Kulkarni 2021), licensed under CC BY 4.0.

**Figure 8:**
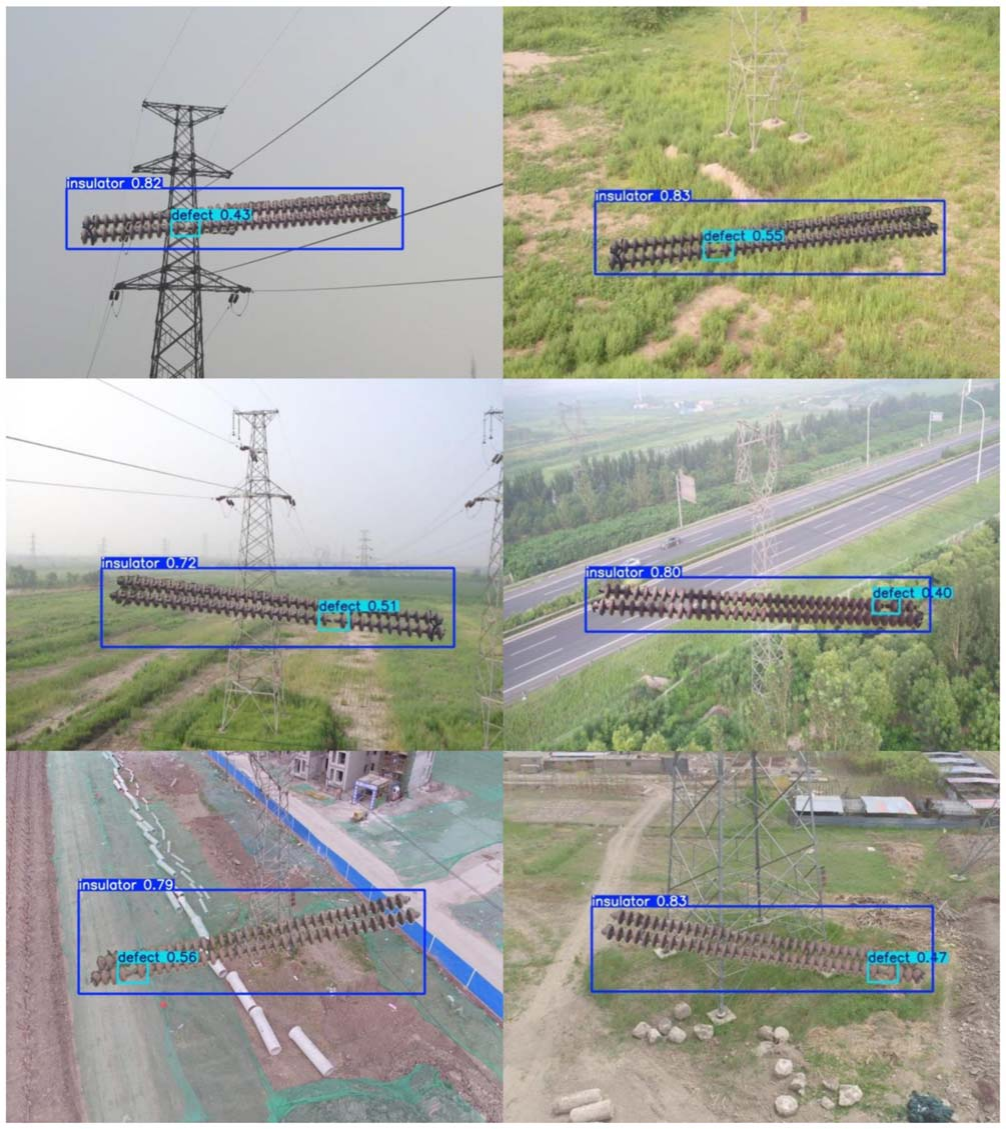
Successful detections on the CPLID dataset on TE-YOLOv8. Input images reproduced from Liu et al. (2024), licensed under CC BY 4.0.

**Figure 9:**
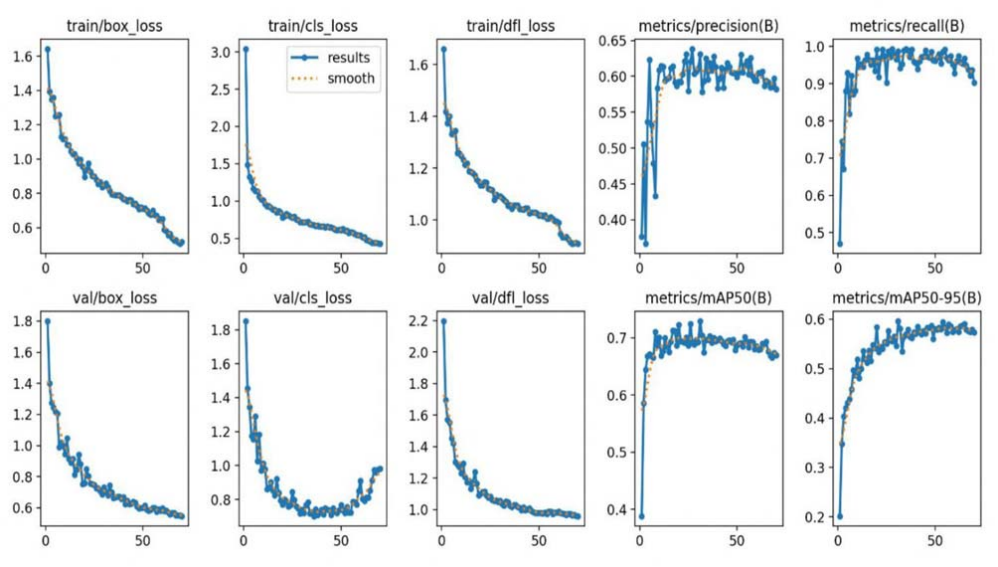
Precision, recall, and mAP as TE-YOLOv8 training progress over the CPLID dataset.

**Figure 10:**
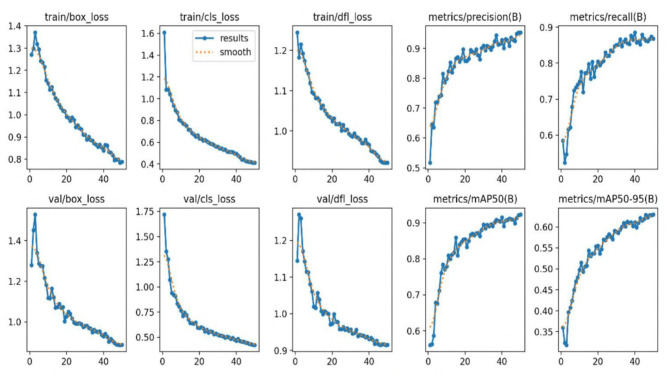
Precision, recall, and mAP as TE-YOLOv8 training progress over the IDID dataset.

Figure 7 illustrates successful detections on the IDID dataset, while Figure 8 shows successful detections on the CPLID dataset using TE-YOLOv8. Additionally, Figures 9, 10 track the progression of performance metrics such as training loss, validation loss, precision, recall, and mAP scores over the course of training. As the number of training epochs increases, both training and validation losses decrease, indicating that the model is improving. Concurrently, metrics like precision, recall, mAP@0.5, and mAP@0.5:0.95 increase, demonstrating enhanced detection capabilities. These results highlight the model's ability to effectively learn and improve over time.

**Figure 11:**
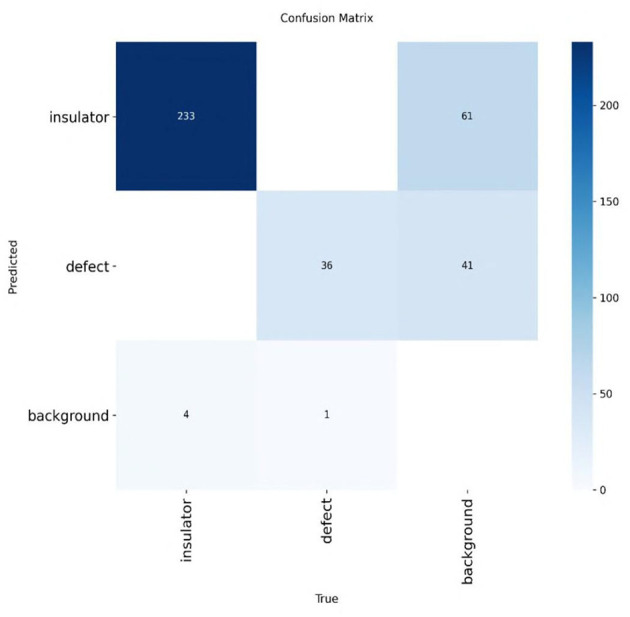
Confusion matrix on the CPLID dataset on TE-YOLOv8.

**Figure 12:**
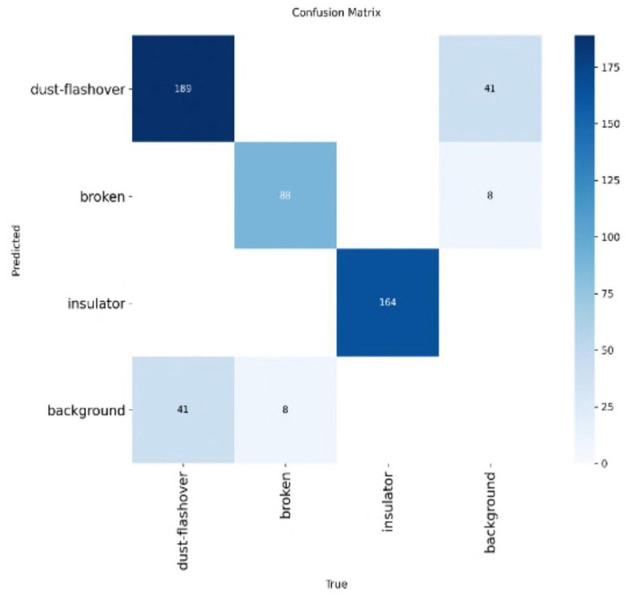
Confusion matrix on the IDID dataset on TE-YOLOv8.

Moreover, Figures 11, 12 present confusion matrices comparing the performance of TE-YOLOv8 against traditional detection models. The results clearly demonstrate that our method outperforms conventional models, underscoring its potential for further advancements in defect detection research.

### 4.3 Discussion of failure cases of TE-YOLOv8

**Figure 13:**
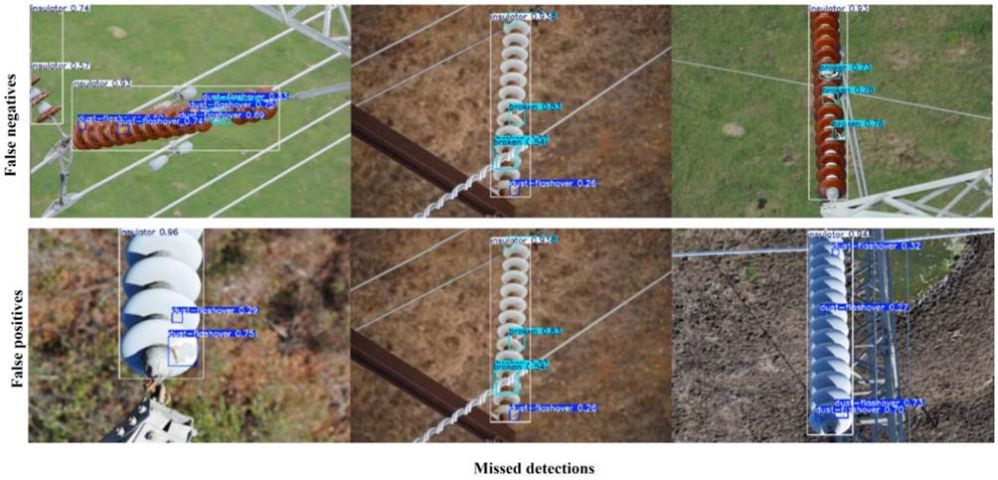
Representative failure cases on the IDID dataset. Input images reproduced from (Lewis and Kulkarni 2021), licensed under CC BY 4.0.

**Figure 14:**
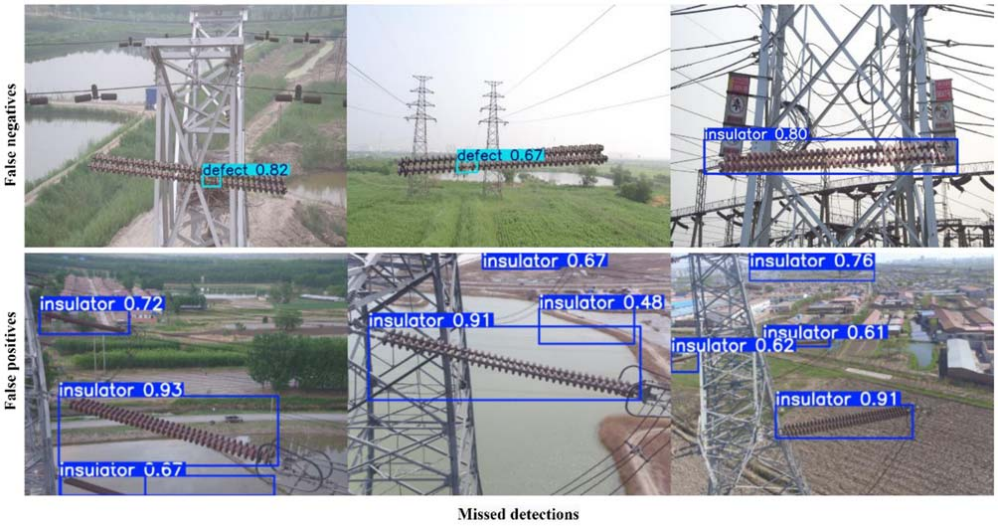
Representative failure cases on the CPLID dataset. Input images reproduced from Liu et al. (2024), licensed under CC BY 4.0.

Figures 13, 14 present representative failure cases of TE-YOLOv8 evaluated on the IDID and CPLID datasets, respectively. As shown in Figure 13 (IDID), the model occasionally produced false negatives, where insulator defects were missed under severe occlusion or when cracks appeared faint, low-contrast, or partially blended into the surface. False positives were also observed, particularly when background structures such as clamps, stains, or shadows resembled defect patterns and were incorrectly classified as faults. Similarly, Figure 14 (CPLID) illustrates failure cases in which defects were overlooked due to extreme scale variation (false negatives), as well as instances where normal insulators were incorrectly flagged as defective. These false positives often arose from background clutter, unusual lighting conditions, or reflections that created visual artifacts mimicking real damage. In both datasets, mislocalized bounding boxes were also observed, especially for elongated insulators or small defect regions.

These examples highlight the remaining challenges for practical deployment of TE-YOLOv8 in real-world inspection scenarios. They emphasize the need for further refinement of the detection framework, particularly in handling low-contrast defects, occlusion, and background interference. Future work will focus on domain adaptation to improve generalization across diverse environments and semi-supervised learning strategies to enhance robustness when annotated data are limited.

### 4.4 Ablation studies

Comprehensive ablation experiments were conducted to quantify the individual and cumulative contributions of the proposed modules in TE-YOLOv8. We progressively integrated the Global Convolution (GConv), C2f-Global Pooling Fusion (C3-GPF), Multiscale Information Fusion (MSIF), Weighted Feature Information Fusion (WFIF), and the transformer-enhanced neck, starting from the YOLOv8 baseline, and measured changes in precision, recall, F1-score, mAP, parameters, and FPS. The results on IDID and CPLID (Tables 4, 5) demonstrate consistent, stepwise gains in detection accuracy with modest computational overhead, confirming that each component contributes meaningfully to performance.

#### 4.4.1 Ablation results on IDID dataset

In Table 8, on the IDID dataset, the baseline YOLOv8 achieved an mAP of 88.7% with 95 FPS and 11.2M parameters. Incorporating the GConv module improved mAP to 90.1% while maintaining high efficiency, demonstrating the benefit of large-kernel decomposed convolutions for capturing extended spatial context. Adding the C2f-GPF module further increased mAP to 91.3%, highlighting the role of global pooling-based recalibration in enhancing discriminative feature extraction. The integration of MSIF yielded 92.6% mAP, confirming the effectiveness of adaptive multi-scale fusion for handling extreme scale variation. With WFIF, performance rose to 93.2% mAP, showing the value of channel-wise attention in suppressing background interference. Finally, the transformer-enhanced neck produced the full TE-YOLOv8 configuration, achieving 94.2% mAP with precision of 95.3% and recall of 93.1%, while maintaining real-time inference at 82 FPS with 14.9M parameters.

**Table 8:** Ablation study results on IDID dataset.

**Model**	**GConv**	**C3f-GPF**	**MSIF**	**WFIF**	**TE-YOLOv8**	**Precision**	**Recall**	**F1**	**mAP**	**Params**	**FPS**
YOLOv8						89.1	89.7	90.2	88.7	11.2M	95
	✓					91.3	91.6	90.7	90.1	11.7M	92
	✓	✓				92.1	92.4	91.9	91.3	12.5M	89
	✓	✓	✓			93.3	92.9	93.1	92.6	13.2M	86
	✓	✓	✓	✓		92.5	92.7	92.4	93.2	13.8M	84
	✓	✓	✓	✓	✓	**95.3**	**93.1**	**93.8**	**94.2**	**14.9M**	**82**

The bold value shows the best results.

#### 4.4.2 Ablation results on CPLID dataset

In Table 9, on the CPLID dataset, similar trends were observed. The baseline YOLOv8 achieved 89.3% mAP with 95 FPS and 11.2M parameters. The addition of GConv improved mAP to 90.8%, reflecting its robustness under challenging atmospheric conditions. Incorporating C3-GPF raised mAP to 91.9%, particularly benefiting detection under hazy and foggy scenarios where global context is critical. The MSIF module further enhanced mAP to 93.1%, validating its ability to integrate features across diverse scales. WFIF contributed an additional gain, bringing mAP to 93.8% with balanced precision and recall, while the transformer neck consolidated these improvements, yielding a final TE-YOLOv8 performance of 93.8% mAP, precision of 94.7%, and F1-score of 94.1% at 82 FPS with 14.9M parameters.

**Table 9:** Ablation study results on CPLID dataset.

**Model**	**GConv**	**C3f-GPF**	**MSIF**	**WFIF**	**TE-YOLOv8**	**Precision**	**Recall**	**F1**	**mAP**	**Params**	**FPS**
YOLOv8						90.2	89.3	89.1	89.3	11.2M	95
	✓					92.1	91.7	92.2	90.8	11.7M	92
	✓	✓				91.1	92.1	92.3	91.9	12.5M	89
	✓	✓	✓			93.4	93.9	91.1	93.1	13.2M	86
	✓	✓	✓	✓		93.9	92.7	93.4	93.8	13.8M	84
	✓	✓	✓	✓	✓	**94.7**	**92.9**	**94.1**	**93.8**	**14.9 M**	**82**

The bold value shows the best results.

Overall, the ablation studies confirm that each proposed module contributes measurable improvements in accuracy while maintaining real-time efficiency. The cumulative gains of +5.5 mAP points on IDID and +4.5 mAP points on CPLID demonstrate the robustness and generalization ability of TE-YOLOv8. Moreover, the varying contribution ratios across datasets reflect their unique challenges: CPLID benefits more from global context modeling under atmospheric degradation, whereas IDID gains more from extended receptive fields and channel-wise prioritization in cluttered backgrounds.

## 5 Conclusion

This research introduces TE-YOLOv8, a novel deep learning framework for automated insulator defect detection in high-voltage transmission systems. By combining transformer-based attention mechanisms with advanced convolutional modules, TE-YOLOv8 achieves superior detection performance while maintaining real-time processing capabilities for UAV-based inspection applications. Key innovations include Global Convolution modules for extended spatial context, C3f-Global Pooling Fusion modules for feature amplification, Multiscale Information Fusion modules for adaptive multi-scale detection, Weighted Feature Information Fusion modules for dynamic channel-wise attention, and a transformer-enhanced neck architecture for global dependency modeling. These modules address critical challenges such as small defect detection, complex backgrounds, scale variations, and adverse imaging conditions. Comprehensive validation on the IDID and CPLID datasets shows TE-YOLOv8 achieves mAPs of 94.2% and 93.8%, respectively, with a 4.9% and 5.1% improvement over baseline YOLOv8, while maintaining real-time inference at 82 FPS. Ablation studies and comparative analyses confirm the model's superiority, establishing a robust foundation for automated smart grid maintenance. Future work could explore multi-modal sensor fusion, detailed defect severity assessment, and active vision strategies to further enhance defect detection capabilities.

### 5.1 Limitations and future directions

Despite its promising performance, several limitations must be addressed. First, the current framework is focused exclusively on visible defects in optical imagery. Incorporating multi-modal fusion, such as infrared thermography or ultraviolet corona imaging, could help detect internal degradation and electrical tracking. Second, current datasets employ coarse classification, and finer taxonomies could enable more detailed severity assessments and failure mechanism identification. Third, few-shot learning techniques or synthetic data augmentation could address the challenge of limited training examples for rare defect categories. Finally, integrating the framework with active vision systems for adaptive image capture upon defect detection could improve inspection efficiency and reliability.

## Data Availability

The datasets presented in this study can be found in online repositories. The names of the repository/repositories and accession number(s) can be found in the article/supplementary material.

## Appendix

**Table A1:** Appendix A: nomenclature.

**Symbol**	**Description**
**F**	Feature map
*C*	Number of feature channels
*H*	Feature map height
*W*	Feature map width
*k*	Convolution kernel size
**W**	Weight matrix
σ	Activation function
*	Convolution operation
⊙	Element-wise multiplication
ℓ	Feature pyramid level
**Q**, **K**, **V**	Query, key, value matrices
*d*_*k*_	Key dimension in attention
η	Learning rate
λ	Loss weighting coefficient
TP	True positives
FP	False positives
FN	False negatives
mAP	Mean average precision
FPS	Frames per second
IoU	Intersection over Union
SIoU	SCYLLA Intersection over Union
GConv	Global Convolution
C3-GPF	C3-Global Pooling Fusion
MSIF	Multiscale Information Fusion
WFIF	Weighted Feature Information Fusion
UAV	Unmanned Aerial Vehicle
CNN	Convolutional Neural Network
YOLO	You Only Look Once
FPN	Feature Pyramid Network
PANet	Path Aggregation Network
BiFPN	Bidirectional Feature Pyramid Network

## References

[B1] Bochkovskiy A. Wang C.-Y. Liao H.-Y. M. (2020). YOLOv4: optimal speed and accuracy of object detection. arXiv:2004.10934. doi: 10.48550/arXiv.2004.10934

[B2] Chen L.-C. Papandreou G. Kokkinos I. Murphy K. Yuille A. L. (2017). DeepLab: semantic image segmentation with deep convolutional nets, atrous convolution, and fully connected CRFs. IEEE Trans. Pattern Anal. Mach. Intell. 40, 834–848. doi: 10.1109/TPAMI.2017.2699184 28463186

[B3] Elfwing S. Uchibe E. Doya K. (2018). Sigmoid-weighted linear units for neural network function approximation in reinforcement learning. Neural Netw. 107, 3–11. doi: 10.1016/j.neunet.2017.12.012 29395652

[B4] Farooq U. Yang F. Shahzadi M. Ali U. Li Z. (2025). YOLOv8-IDX: optimized deep learning model for transmission line insulator-defect detection. Electronics 14:1828. doi: 10.3390/electronics14091828

[B5] Gevorgyan Z. (2022). SIoU loss: more powerful learning for bounding box regression. arXiv:2205.12740. doi: 10.48550/arXiv.2205.12740

[B6] Girshick R. (2015). “Fast R-CNN,” in 2015 IEEE International Conference on Computer Vision (ICCV) (Santiago: IEEE), 1440–1448. doi: 10.1109/ICCV.2015.169

[B7] Girshick R. Donahue J. Darrell T. Malik J. (2014). “Rich feature hierarchies for accurate object detection and semantic segmentation,” in 2014 IEEE Conference on Computer Vision and Pattern Recognition (Columbus, OH: IEEE), 580–587. doi: 10.1109/CVPR.2014.81

[B8] Hao K. Chen G. Zhao L. Li Z. Liu Y. Wang C. (2022). An insulator defect detection model in aerial images based on multiscale feature pyramid network. IEEE Trans. Instrum. Meas. 71, 1–12. doi: 10.1109/TIM.2022.3200861

[B9] He K. Zhang X. Ren S. Sun J. (2016). “Deep residual learning for image recognition,” in 2016 IEEE Conference on Computer Vision and Pattern Recognition (CVPR) (Las Vegas, NV: IEEE), 770–778. doi: 10.1109/CVPR.2016.90

[B10] Hu M. Liu J. Liu J. (2025). DRR-YOLO: a study of small target multi-modal defect detection for multiple types of insulators based on large convolution kernel. IEEE Access 13:3539831. doi: 10.1109/ACCESS.2025.3539831

[B11] Hu Z. Zhai B. Zhao Z. Zhai Y. Wang Q. Yang K. (2025). State-space-model-guided deep feature perception network for insulator defect detection in high-resolution aerial images. IEEE Trans. Geosci. Remote Sens. 63:3584663. doi: 10.1109/TGRS.2025.3584663

[B12] Huang L. Li Y. Wang W. He Z. (2023). Enhanced detection of subway insulator defects based on improved YOLOv8. Appl. Sci. 13:13044. doi: 10.3390/app132413044

[B13] Huang S. Dong X. Wang Y. Yang L. (2022). “Detection of insulator burst position of lightweight YOLOv8,” in ICCAI '22: Proceedings of the 8th International Conference on Computing and Artificial Intelligence (Tianjin: Association for Computing Machinery), 573–578. doi: 10.1145/3532213.3532300

[B14] Lewis D. Kulkarni P. (2021). Insulator Defect Detection Dataset (IDID). IEEE Dataport.

[B15] Li C. Shi Y. Lu M. Zhou S. Xie C. Chen Y. (2025). A composite insulator overheating defect detection system based on infrared image object detection. IEEE Trans. Power Del. 40:3488061. doi: 10.1109/TPWRD.2024.3488061

[B16] Li C. Wang L. Zhang Y. Weng K. Geng Y. Li L. . (2022). YOLOv6: a single-stage object detection framework for industrial applications. arXiv:2209.02976. doi: 10.48550/arXiv.2209.02976

[B17] Li D. Hu J. Wang C. Li X. She Q. Zhu L. . (2021). “Involution: inverting the inherence of convolution for visual recognition,” in 2021 IEEE/CVF Conference on Computer Vision and Pattern Recognition (CVPR) (Nashville, TN: IEEE), 12316–12325. doi: 10.1109/CVPR46437.2021.01214

[B18] Li J. Zhou H. Lv G. Chen J. (2025). A2MADA-YOLO: attention alignment multiscale adversarial domain adaptation YOLO for insulator defect detection in generalized foggy scenario. IEEE Trans. Instrum. Meas. 74:3541814. doi: 10.1109/TIM.2025.3541814

[B19] Lin T.-Y. Dollár P. Girshick R. He K. Hariharan B. Belongie S. (2017). “Feature pyramid networks for object detection,” in 2017 IEEE Conference on Computer Vision and Pattern Recognition (CVPR) (Honolulu, HI: IEEE), 936–944. doi: 10.1109/CVPR.2017.106

[B20] Liu C. Wu Y. Liu J. Han J. (2021). MTI-YOLO: a light-weight and real-time deep neural network for insulator detection in complex aerial images. Energies 14:1426. doi: 10.3390/en14051426

[B21] Liu J. Ou Y. Zhou Z. Jiao R. Wu T. (2025). Lightweight method for insulator defect detection based on improved convolutional neural networks. IEEE Trans. Instrum. Meas. 74:3599701. doi: 10.1109/TIM.2025.3599701

[B22] Liu Q. Liu Y. Yan Y. Jiang Q. Jiang X. (2025). Addressing domain shift in insulator defect data: a generalization framework for cross-domain detection of broken and self-blast insulator defect. IEEE Trans. Instrum. Meas. 74:3580815. doi: 10.1109/TIM.2025.3580815

[B23] Liu W. Anguelov D. Erhan D. Szegedy C. Reed S. Fu C.-Y. Berg A. C. (2016). “SSD: single shot MultiBox detector,” in Proc. Eur. Conf. Comput. Vis. (ECCV) (Cham: Springer), pp. 21–37. doi: 10.1007/978-3-319-46448-0_2

[B24] Liu Y. Zhang X. Wang J. (2024) Chinese Power Line Insulator Dataset (CPLID). IEEE DataPort.

[B25] Lu G. Li B. Chen Y. Qu S. Cheng T. Zhou J. (2025). Precision in aerial surveillance: integrating YOLOv8 with PConv and CoT for accurate insulator defect detection. IEEE Access 13:3551289. doi: 10.1109/ACCESS.2025.3551289

[B26] Ma W. Wang B. Zhao Z. Wang Q. Chen B. (2025). A small-sized defect detection method for power line insulator using multiscale feature and lightweight networks in UAV-vision. IEEE Trans. Power Del. 40:3589542. doi: 10.1109/TPWRD.2025.3589542

[B27] Ou J. Wang J. Xue J. Wang J. (2023). Infrared image target detection of substation electrical equipment using an improved faster R-CNN. IEEE Trans. Power Del. 38, 387–396. doi: 10.1109/TPWRD.2022.3191694

[B28] Redmon J. Divvala S. Girshick R. Farhadi A. (2016). “You only look once: unified, real-time object detection,” in IEEE Conference on Computer Vision and Pattern Recognition (CVPR) (Las Vegas, NV: IEEE), 779–788. doi: 10.1109/CVPR.2016.91

[B29] Redmon J. Farhadi A. (2018). YOLOv3: an incremental improvement. arXiv:1804.02767. doi: 10.48550/arXiv.1804.02767

[B30] Ren S. He K. Girshick R. Sun J. (2015). Faster R-CNN: towards real-time object detection with region proposal networks. Proc. Adv. Neural Inf. Process. Syst. 28, 1–9. doi: 10.1109/TPAMI.2016.2577031 27295650

[B31] Shaikh J. A. Wang C. Saifullah S.ima, M. W. U. Arshad M. Rathore W. U. A. (2025). Memory feedback transformer based intrusion detection system for IoMT healthcare networks. Internet Things 32:101597. doi: 10.1016/j.iot.2025.101597

[B32] Shen P. Mei K. Cao H. Zhao Y. Zhang G. (2025). LDDFSF-YOLO11: a lightweight insulator defect detection method focusing on small-sized features. IEEE Access 13:3569970. doi: 10.1109/ACCESS.2025.3569970

[B33] Shuang F. Wei S. Li Y. Gu X. Lu Z. (2023). Detail R-CNN: insulator detection based on detail feature enhancement and metric learning. IEEE Trans. Instrum. Meas. 72, 1–14. doi: 10.1109/TIM.2023.330566737323850

[B34] Souza B. J. Stefenon S. F. Singh G. Freire R. Z. (2023). HybrTE-YOLOv8 for classification of insulators defects in transmission lines based on UAV. Int. J. Electr. Power Energy Syst. 148:108982. doi: 10.1016/j.ijepes.2023.108982

[B35] Tan M. Pang R. Le Q. V. (2020). “EfficientDet: scalable and efficient object detection,” in 2020 IEEE/CVF Conference on Computer Vision and Pattern Recognition (CVPR) (Seattle, WA: IEEE), 10778–10787. doi: 10.1109/CVPR42600.2020.01079

[B36] Vaswani A. Shazeer N. Parmar N. Uszkoreit J. Jones L. Gomez A. N. . (2017). Attention is all you need. arXiv preprint arXiv:1706.03762. doi: 10.48550/arXiv.1706.03762

[B37] Wang C.-Y. Bochkovskiy A. Liao H.-Y.-M. (2023). “YOLOv7: trainable bag-of-freebies sets new state-of-the-art for real-time object detectors,” in 2023 IEEE/CVF Conference on Computer Vision and Pattern Recognition (CVPR) (Vancouver, BC: IEEE), 7464–7475. doi: 10.1109/CVPR52729.2023.00721

[B38] Wang C.-Y. Liao H.-Y. M. Wu Y.-H. Chen P.-Y. Hsieh J.-W. Yeh I.-H. (2020). “CSPNet: a new backbone that can enhance learning capability of CNN,” in 2020 IEEE/CVF Conference on Computer Vision and Pattern Recognition Workshops (CVPRW) (Seattle, WA: IEEE), 390–391. doi: 10.1109/CVPRW50498.2020.00203

[B39] Wang Y. Qu Z. Hu Z. Yang C. Huang X. Zhao Z. . (2025). Cross-domain multilevel feature adaptive alignment R-CNN for insulator defect detection in transmission lines. IEEE Trans. Instrum. Meas. 74:3527619. doi: 10.1109/TIM.2025.3527619

[B40] Wang Z. Liu X. Peng H. Zheng L. Gao J. Bao Y. (2021). Railway insulator detection based on adaptive cascaded convolutional neural network. IEEE Access 9, 115676–115686. doi: 10.1109/ACCESS.2021.3105419

[B41] Wei Z. Wei Y. (2025). YOLOv10n-based defect detection in power insulators: attention enhancement and feature fusion optimization. IEEE Access 13:3581672. doi: 10.1109/ACCESS.2025.3581672

[B42] Woo S. Park J. Lee J. Kweon I. S. (2018). “CBAM: convolutional block attention module,” in Proc. Eur. Conf. Comput. Vis. (ECCV) (Cham: Springer), 3–19. doi: 10.1007/978-3-030-01234-2_1

[B43] Xu J. Liao H. Li K. Jiang C. Li D. (2025). Multiscale feature fusion transformer with hybrid attention for insulator defect detection. IEEE Trans. Instrum. Meas. 74:3568984. doi: 10.1109/TIM.2025.3568984

[B44] Yang Y. Wang X. (2023). Insulator detection using small samples based on YOLOv8 in natural background. Multimedia Tools Appl. 82, 44841–44857. doi: 10.1007/s11042-023-15722-1

[B45] Yang Z. Xu Z. Wang Y. (2022). Bidirection-fusion-YOLOv3: An improved method for insulator defect detection using UAV image. IEEE Trans. Instrum. Meas. 71, 1–8. doi: 10.1109/TIM.2022.3201499

[B46] Yu F. Koltun V. (2015). Multi-scale context aggregation by dilated convolutions. arXiv:1511.07122. doi: 10.48550/arXiv.1511.07122

[B47] Yu K. Zhao L. Xue X. Li H. Liu H. (2025). SMA-YOLO: a defect detection algorithm for self-explosion of insulators under complex backgrounds. IEEE Access 13:3609906. doi: 10.1109/ACCESS.2025.3609906

[B48] Yu Z. Lei Y. Shen F. Zhou S. Yuan Y. (2023). Research on identification and detection of transmission line insulator defects based on a lightweight YOLOv8 network. Remote Sens. 15:4552. doi: 10.3390/rs15184552

[B49] Yuan P. Pu Y. Liu C. (2021). Improving electricity supply reliability in China: cost and incentive regulation. Energy 237:121558. doi: 10.1016/j.energy.2021.121558

[B50] Zhang Q. Zhang J. Li Y. Zhu C. Wang G. (2025). TE-YOLOV8: a multimodule optimized algorithm for insulator defect detection in power transmission lines. IEEE Trans. Instrum. Meas. 74:3527530. doi: 10.1109/TIM.2025.3527530

[B51] Zhang T. Zhang Y. Xin M. Liao J. Xie Q. (2023). A light-weight network for small insulator and defect detection using UAV imaging based on improved YOLOv8. Sensors 23:5249. doi: 10.3390/s23115249 37299976 PMC10256046

[B52] Zhang Y. Wang B. Yang Q. Tang F. Wei K. (2025). A two-stage insulator defect detection network with sequence transduction. IEEE Trans. Instrum. Meas. 74:3522390. doi: 10.1109/TIM.2024.3522390

[B53] Zhou M. Wang J. Li B. (2022). ARG-mask RCNN: an infrared insulator fault-detection network based on improved mask RCNN. Sensors 22:4720. doi: 10.3390/s22134720 35808217 PMC9268765

